# Efficacy and Safety of Plasma Radiofrequency Combined With Topical Tranexamic Acid for Early Post‐Traumatic Scars With Hyperpigmentation: A Retrospective Study

**DOI:** 10.1111/jocd.70848

**Published:** 2026-04-09

**Authors:** Yue Liu, Mingtong Fang, Yuanyuan Xu, Meng Wang, Maomei Dou, Yuchen Zhang, Shasha Li, Jing Dou, Wei Zhang, Lianzhao Wang

**Affiliations:** ^1^ Department of Cicatrix Minimally Invasive Treatment Center, Plastic Surgery Hospital, Chinese Academy of Medical Sciences and Peking Union Medical College Beijing China

**Keywords:** combination therapy, plasma radiofrequency, post‐inflammatory hyperpigmentation, scar, tranexamic acid

## Abstract

**Purpose:**

Treatment of early post‐traumatic scars with energy‐based devices like plasma radiofrequency is often complicated by post‐inflammatory hyperpigmentation (PIH). This study aimed to evaluate the efficacy and safety of a combination regimen of plasma radiofrequency and immediate topical application of tranexamic acid (TXA) to simultaneously address both scars and concomitant PIH.

**Methods:**

This retrospective study included 35 patients with early post‐traumatic scars and hyperpigmentation. All patients underwent three sessions of plasma radiofrequency at 6‐week intervals, each followed by immediate in‐office and 7‐day at‐home topical application of TXA. Efficacy was evaluated using the Modified Vancouver Scar Scale (mVSS), Investigator's Global Assessment (IGA), and a patient‐reported Visual Analogue Scale (VAS) for satisfaction at baseline and 6 weeks after the final session.

**Results:**

Significant improvements were observed in all patients. The mean mVSS score decreased from a baseline of 5.71 ± 1.47 to 2.66 ± 2.07 at the final follow‐up (*p* < 0.001). According to IGA, 91.4% (32/35) of patients achieved moderate to excellent improvement. High patient satisfaction was reported on the VAS. No serious adverse events were observed; side effects were limited to transient erythema and edema.

**Conclusion:**

The combination of plasma radiofrequency with immediate topical TXA application is a safe and effective strategy for treating early scars with hyperpigmentation. This dual‐action approach successfully improves scar characteristics while controlling and reversing pigmentation, offering a valuable therapeutic option to optimize outcomes with energy‐based devices.

## 1 Introduction

The clinical management of early post‐traumatic scars presents a dual challenge: remodeling abnormal collagen while controlling the frequently associated post‐inflammatory hyperpigmentation (PIH) [1, 2]. Energy‐based devices (EBDs), such as plasma radiofrequency, are established modalities for scar remodeling [3]. However, their inherent inflammatory stimulus poses a significant clinical dilemma, as it can paradoxically trigger or worsen PIH.

This paradox was strikingly illustrated in our initial clinical experience with three patients. For their first treatment session, they received plasma radiofrequency followed by our standard postoperative care (including an anti‐inflammatory ointment). The outcome was suboptimal: hyperpigmentation failed to improve, and notably, one patient experienced a significant exacerbation of her PIH.

This adverse event prompted an immediate strategy change. For their subsequent three sessions, we incorporated immediate topical application of tranexamic acid (TXA). The results were transformative: a consistent session‐by‐session improvement in pigmentation was observed in all three patients.

This powerful N‐of‐3, sequentially controlled observation strongly suggested that TXA was the critical missing element for pigment control. It led us to hypothesize that a synergistic effect could be achieved: plasma for physical remodeling and drug delivery, and TXA for targeted biochemical inhibition [4, 5].

Therefore, prompted by these powerful initial outcomes, we conducted this retrospective study to systematically evaluate the efficacy and safety of this dual‐action strategy in a larger patient cohort.

## 2 Materials and Methods

### 2.1 Study Design and Ethics

This single‐center, retrospective study was conducted at the Hospital. The study protocol was approved by the Institutional Review Board (IRB) of our institution. The study was conducted in accordance with the principles of the Declaration of Helsinki. Written informed consent was obtained from all patients before any procedure, covering the treatment itself, photography, and the potential use of their anonymized data for research purposes.

### 2.2 Pilot Case Series

Before the main cohort, three patients with early post‐traumatic scars and PIH were managed. For their first session, they received plasma radiofrequency followed by standard postoperative care (including Longzhu ointment). Due to unsatisfactory pigmentary outcomes, including one case of exacerbation, the protocol for their subsequent three treatment sessions was modified to include the immediate topical application of TXA. The successful outcomes of these cases prompted the retrospective analysis of the larger cohort.

### 2.3 Study Population for Retrospective Analysis

A total of 35 patients who received the final combination treatment protocol between January 1, 2023, and December 31, 2024, were included in this retrospective analysis.

#### 2.3.1 Inclusion Criteria

(1) Age 18–60 years, any gender; (2) Early post‐traumatic scar (1–6 months duration) on the face or trunk; (3) Concomitant PIH; (4) Fitzpatrick skin type III‐IV; (5) Availability of complete medical records and standardized clinical photographs.

#### 2.3.2 Exclusion Criteria

(1) Personal or family history of keloids or hypertrophic scarring; (2) Known allergy to TXA or topical anesthetics; (3) Active infection in the treatment area; (4) Other scar treatments within the past 6 months; (5) Pregnancy or lactation.

### 2.4 Treatment Protocol for the Main Cohort

#### 2.4.1 Pre‐Treatment Preparation

The treatment area was cleansed, and a topical compound lidocaine cream (Beijing Tsinghua Ziguang Pharmaceutical Factory, Beijing, China) was applied under occlusion for 40–60 min. Standardized photographs were taken using a DSLR camera under consistent lighting, angle, and distance.

#### 2.4.2 Plasma Radiofrequency Treatment

A medical radiofrequency system (Legato II, Alma Lasers, Caesarea, Israel) was used with a 3‐row Roller‐Tip in Micro Plasma—IN Motion mode. The settings were: ABLATIVE level 2, with a power of 8‐10 W. The handpiece was passed over the scar area for 3 uniform passes at a speed of approximately 6 cm/s. The clinical endpoint was mild, uniform erythema.

#### 2.4.3 Combination Therapy and Post‐Procedure Care

##### 2.4.3.1 In‐office application

Immediately after the plasma treatment, a sterile gauze soaked in pharmaceutical‐grade tranexamic acid solution for injection (5 mL:0.25 g, CHENGDU FIRST PHARMACEUTICAL, Chengdu, China) was applied as a wet compress to the treated area for 20–30 min. Following the compress, a layer of Longzhu ointment (Mayinglong Pharmaceutical Group Co. LTD, Wuhan, China), approximately 1 mm thick, was applied over the treated area, which was then covered with a sterile gauze dressing for protection. According to its official product description, this traditional herbal‐based ointment possesses anti‐inflammatory and wound‐healing properties.

##### 2.4.3.2 At‐home care

Patients were instructed on a twice‐daily at‐home care regimen. First, they applied the tranexamic acid solution to the treated area using a sterile cotton swab. After its absorption, a layer of Longzhu ointment, approximately 1 mm thick, was applied. The area was then protected with a fresh sterile gauze dressing to shield the healing tissue from external contact and friction.

This regimen was continued until the micro‐crusts from the treatment had fully shed and the skin had re‐epithelialized, a process that typically took approximately 7 days. Patients were instructed to continue this care protocol if desquamation was delayed. This intensive post‐procedure care was designed to support the critical phase of wound healing and control inflammation until the skin barrier was restored.

##### 2.4.3.3 Long‐Term Scar Management

After complete re‐epithelialization and desquamation (typically around day 7), patients were instructed to begin long‐term scar management. This consisted of applying a self‐adherent silicone dressing (Mepiform, Mölnlycke Health Care, Gothenburg, Sweden) over the treated scar. Patients were advised to wear the silicone dressing for a minimum of 20 h per day throughout the 6‐week intervals between treatment sessions and to continue for at least 3 months after the final session.

#### 2.4.4 Treatment Course

A complete course of treatment consisted of a total of three sessions for each patient, with a 6‐week interval between sessions. The final follow‐up and outcome assessment were performed 6 weeks after the last treatment session.

### 2.5 Efficacy and Safety Assessment

Efficacy was evaluated by two experienced dermatologists who were blinded to the treatment sessions and did not perform the procedures.

(1) Investigator's Global Assessment (IGA):

Standardized photographs from baseline and the final follow‐up were compared and graded on a 5‐point scale: 4 = Excellent improvement (> 75%), 3 = Moderate improvement (51%–75%), 2 = Slight improvement (26%–50%), 1 = No change (0%–25%), or 0 = Worsening.

(2) Modified Vancouver Scar Scale (mVSS):

Scars were assessed for vascularity, pigmentation, pliability, and height, with a total score ranging from 0 to 13.

(3) Patient Satisfaction:

A 10‐point Visual Analogue Scale (VAS) was used, where 0 indicated “completely dissatisfied” and 10 indicated “extremely satisfied”.

(4) Safety Assessment:

Adverse events, including pain, erythema, edema, infection, and any changes in pigmentation, were recorded at each visit.

### 2.6 Statistical Analysis

Statistical analysis was performed using SPSS software (Version 26.0, IBM Corp., Armonk, NY, USA). Continuous data were expressed as mean ± standard deviation (SD). The normality of data distribution was assessed using the Shapiro–Wilk test. For normally distributed data (e.g., mVSS, VAS scores), the paired samples *t*‐test was used to compare values before and after treatment. For ordinal data (e.g., IGA scores), results were presented as frequencies and percentages. A P‐value < 0.05 was considered statistically significant.

## 3 Results

### 3.1 Outcomes of the Pilot Case Series

**FIGURE 1:**
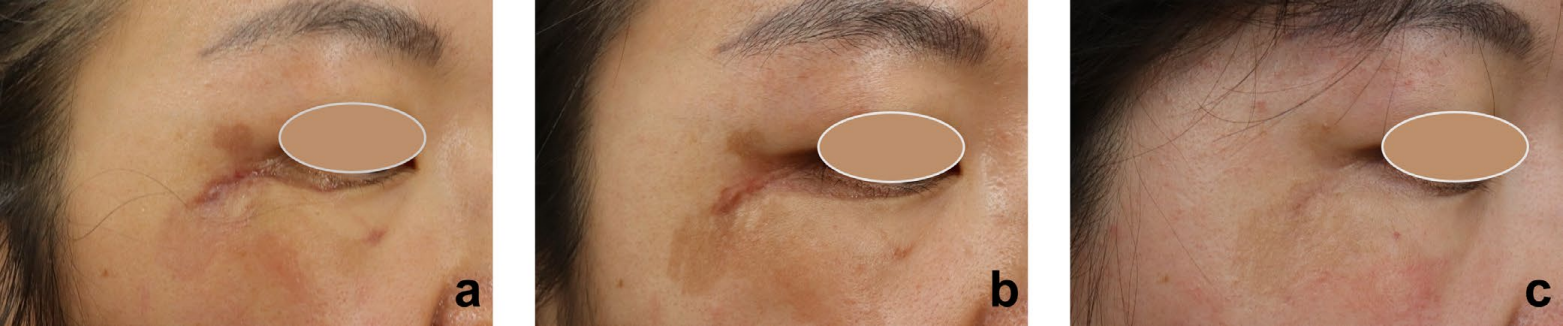
Representative case from the pilot series illustrating the pivotal role of topical tranexamic acid (TXA). (a) Baseline photograph of an early post‐traumatic scar with significant post‐inflammatory hyperpigmentation (PIH). (b) Six weeks after the first session of plasma radiofrequency combined with standard postoperative care (without TXA), showing marked exacerbation of PIH. (c) Six weeks after the final (fourth) treatment session, which included three sessions with the addition of topical TXA, demonstrating significant resolution of both the PIH and the scar.

The three pilot cases provided pivotal evidence for the necessity of TXA. Initially, using only plasma radiofrequency and standard ointment, the PIH failed to improve: the mVSS pigmentation scores remained at 2 in two patients and paradoxically increased from 2 to 3 in the third patient. However, following the introduction of topical TXA in the subsequent three sessions, a dramatic reversal was observed. By the final follow‐up, the pigmentation scores had significantly improved (decreasing to 0 in one patient and to 2 with marked clinical fading in the other two). This sequential observation highlights that the therapeutic success was contingent upon the addition of TXA, as further illustrated by the representative case in Figure 1.

### 3.2 Demographics and Baseline Characteristics of the Retrospective Cohort

A total of 35 patients who received the full combination protocol were included in the retrospective analysis. The cohort consisted of 8 males (22.9%) and 27 females (77.1%), with a mean age of 35.2 ± 10.8 years (range, 18–58). All patients had Fitzpatrick skin types III or IV. The mean scar duration before treatment was 2.4 ± 1.6 months. Scars were located on the face (*n* = 23, 65.7%), extremities (*n* = 8, 22.9%), and trunk (*n* = 4, 11.4%). All patients completed the full course of three treatment sessions and the 6‐week post‐treatment follow‐up. Detailed demographic data are summarized in Table 1.

**TABLE 1:** Demographics and baseline characteristics of the study cohort (*N* = 35).

Characteristic	Value
Age, years (Mean ± SD)	35.2 ± 10.8
Range	18–58
Gender, *n* (%)	
Male	8 (22.9%)
Female	27 (77.1%)
Fitzpatrick Skin Type, *n* (%)	
Type III	15 (42.9%)
Type IV	20 (57.1%)
Scar Duration, months (Mean ± SD)	2.4 ± 1.6
Scar Location, *n* (%)	
Face	23 (65.7%)
Extremities	8 (22.9%)
Trunk	4 (11.4%)

### 3.3 Efficacy Outcomes

#### 3.3.1 Investigator's Global Assessment (IGA)

According to the blinded evaluators' IGA scores, a significant majority of patients achieved substantial improvement. Specifically, 15 patients (42.9%) were rated as having “Excellent improvement” (> 75%), and 17 patients (48.6%) had “Moderate improvement” (51%–75%). The remaining 3 patients (8.6%) showed “Slight improvement” (26%–50%). Overall, 91.4% of patients achieved a moderate to excellent outcome.

#### 3.3.2 Scar Scale Improvement (mVSS)

The combination therapy significantly improved individual scar characteristics. Regarding the pigmentation sub‐score, all 35 patients (100%) scored 2 at baseline. Following treatment, the mean score significantly decreased to 1.14 ± 0.99 (*p* < 0.001), with 15 patients (42.9%) achieving a complete return to normal skin color (score 0). Notably, while 20 patients (57.1%) remained in the “hyperpigmentation” category (score 2) due to the lack of intermediate tiers in the mVSS, a clinically marked lightening of the PIH was observed in all these cases. Similarly, the vascularity sub‐score showed a dramatic reduction from a baseline of 2.0 ± 0.0 (all “Red”) to 1.11 ± 0.68 (*p* < 0.001), with 21 patients (60.0%) improving to “Pink” and 5 (14.3%) to “Normal.” Detailed sub‐score data are summarized in Table 2.

**TABLE 2:** Breakdown of mVSS sub‐scores before and after treatment (*N* = 35).

mVSS Sub‐score	Baseline (Mean ± SD)	Post‐treatment (Mean ± SD)	*P*‐value*
Pigmentation	2.0 ± 0.0	1.14 ± 0.99	< 0.001
Vascularity	2.0 ± 0.0	1.11 ± 0.68	< 0.001
Pliability	0.86 ± 0.73	0.20 ± 0.41	< 0.001
Height	0.86 ± 0.73	0.20 ± 0.41	< 0.001
Total Score	5.71 ± 1.47	2.66 ± 2.07	< 0.001

#### 3.3.3 Patient‐Reported Satisfaction (VAS)

Patients reported high levels of satisfaction with the treatment outcomes. On a 10‐point scale, the mean VAS score for overall efficacy was 9.2 ± 0.5, and the mean VAS score for pigmentation improvement was 9.4 ± 0.6.

### 3.4 Safety and Tolerability

The treatment regimen was well‐tolerated by all 35 patients. The most common adverse effects were transient and mild. Intraoperative pain was reported at a mean VAS score of 2.3 ± 1.5 (on a 0–10 scale). Post‐procedure erythema and mild edema were observed in all patients, resolving spontaneously within 3–5 days without intervention. Notably, no cases of infection, delayed healing, paradoxical hyperpigmentation, or scar progression (hypertrophy or keloid formation) were observed throughout the study period.

## 4 Discussion

This retrospective study suggests that combining plasma radiofrequency with the immediate topical application of TXA is a safe and effective strategy for the concurrent treatment of early post‐traumatic scars and associated PIH. Our findings demonstrate a significant improvement in both the physical characteristics of the scars, as measured by the mVSS, and a marked reduction in pigmentation, supported by high patient‐reported satisfaction scores.

The most compelling evidence for the pivotal role of TXA comes from our pilot case series, which provided a pragmatic, sequentially controlled observation. Initially, all three patients received plasma radiofrequency combined with Longzhu ointment—a standard postoperative treatment in our clinical practice known for its wound‐healing properties. Crucially, in these cases, the PIH failed to improve or even visibly exacerbated after the first session. Since plasma radiofrequency is theoretically designed to minimize thermal damage to the epidermis and is generally considered to have a low risk for inducing pigmentation, this observed exacerbation during the initial “ointment‐only” phase underscores a critical clinical reality: for scars in an early, pigment‐active state, even the most advanced energy‐based devices and standard anti‐inflammatory care may be insufficient to halt progressive melanogenesis.

The subsequent, dramatic reversal of hyperpigmentation occurred only after TXA was introduced to the protocol. This confirms that while the traditional ointment provides the necessary groundwork for skin barrier repair and the plasma device performs physical structural remodeling, TXA acts as the essential biological regulator. It specifically targets the melanogenic pathways, ensuring that the device's therapeutic heat does not inadvertently stimulate hyperactive melanocytes. By effectively “isolating” the TXA variable through this pilot failure and subsequent success, we demonstrate that TXA is not merely an adjuvant but a therapeutic necessity for managing PIH in this high‐risk population.

The success of this combination protocol is rooted in a sophisticated synergistic mechanism. The plasma radiofrequency acts as a “double‐edged sword”: its thermal energy effectively stimulates dermal remodeling to improve scar texture, but this same thermal effect can paradoxically incite the inflammatory cascade that drives PIH [3]. The addition of TXA masterfully resolves this dilemma. Crucially, the micro‐channels created by the plasma serve as a highly efficient drug delivery system, overcoming the stratum corneum barrier and allowing TXA to reach its target melanocytes. Once delivered, TXA acts as a potent “biological regulator,” inhibiting plasmin to reduce inflammation and directly downregulating tyrosinase activity to curb melanin production at its source [4, 5].

Our findings contribute novel insights to the existing literature. Although the efficacy of plasma radiofrequency for scars and TXA for pigmentary disorders is independently well‐documented [6, 7, 8, 9], studies combining them to specifically solve the clinical challenge of preventing and reversing PIH during energy‐based device treatment are scarce. Our study demonstrates not only the feasibility of this combination but, through our pilot cases, its critical necessity, offering a valuable pathway for the safer application of EBDs in susceptible populations (Fitzpatrick skin types III‐IV).

It is important to attribute the final favorable scar outcomes to the comprehensive, multi‐modal protocol used. The treatment pathway initiated collagen remodeling with plasma radiofrequency, controlled acute inflammation and pigmentation with TXA and supportive care, and promoted long‐term scar maturation with silicone sheeting (Mepiform). This integrated approach, which includes a robust post‐procedure and long‐term care regimen, reflects a successful clinical strategy for managing complex traumatic scars.

Nevertheless, this study has several limitations. First, its retrospective nature is subject to inherent selection and information bias. Second, the absence of a parallel, randomized control group means our conclusions on the superiority of the combination therapy are based on strong inference from the pilot cases rather than direct comparative analysis. Consequently, our findings should be interpreted as hypothesis‐generating rather than definitive evidence of causal efficacy. Third, this was a single‐center study with a relatively limited sample size. Finally, the 6‐week post‐treatment follow‐up is relatively short, and the absence of objective pigmentary assessments, such as colorimetry or mMASI scores, represents a significant methodological limitation. Although mVSS and IGA provide clinical insights, they are inherently semi‐quantitative and may not capture subtle chromatic changes as precisely as digital tools.

## 5 Conclusion

The findings of our study strongly suggest that the combination of plasma radiofrequency with immediate topical application of tranexamic acid is a promising, safe, and effective dual‐action strategy for early post‐traumatic scars with concomitant hyperpigmentation. This integrated protocol allows clinicians to confidently utilize the powerful scar remodeling capabilities of an energy‐based device while proactively managing and reversing the challenging complication of PIH. Future large‐scale, multicenter, prospective randomized controlled trials are warranted to provide a higher level of evidence and further validate these encouraging results.

## Author Contributions

Lianzhao Wang and Yue Liu contributed to the study conception and design. Yue Liu, Yuanyuan Xu, Maomei Dou, Shasha Li, Jing Dou, and Wei Zhang performed the data collection and clinical follow‐up. Yue Liu, Mingtong Fang, Meng Wang, and Yuchen Zhang were responsible for data analysis and interpretation. Yue Liu wrote the original draft of the manuscript. Mingtong Fang, Lianzhao Wang, and Yuanyuan Xu provided critical revision of the manuscript for important intellectual content. All authors read and approved the final manuscript. Yue Liu and Mingtong Fang contributed equally to this work.

## Funding

This work was supported by the Plastic Surgery Hospital, Chinese Academy of Medical Sciences and Peking Union Medical College (Grant No. YS2024CG001).

## Ethics Statement

This retrospective study was conducted in accordance with the principles of the Declaration of Helsinki. Approval was granted by the Review Board of Plastic Surgery Hospital, Chinese Academy of Medical Sciences (Approval No.: 2025 Registration No. 214).

## Consent

Written informed consent was obtained from all individual participants included in the study, covering the treatment itself, clinical photography, and the potential use of their anonymized data and images for research and publication purposes. The authors attest to obtaining written patient consent for the publication of recognizable patient photographs, with the understanding that this information may be publicly available.

## Conflicts of Interest

The authors declare no conflicts of interest.

## Data Availability

Research data are not shared.
